# Prevalence of bilateral Discoid Lateral Menisci (DLM) in patients operated for symptomatic DLM with a follow-up study on their asymptomatic contralateral knees: a Magnetic Resonance Imaging (MRI) assessment

**DOI:** 10.1186/s12891-015-0626-y

**Published:** 2015-07-28

**Authors:** Wen-Xin Liu, Jin-Zhong Zhao, Xiao-Qiao Huangfu, Yao-Hua He, Xing-Guang Yang

**Affiliations:** Department of Sports Medicine, Shanghai Jiao Tong University Affiliated Sixth People’s Hospital, Shanghai, 200233 China

**Keywords:** Discoid lateral meniscus, Bilateral, MRI, Arthroscopy, Han Chinese

## Abstract

**Background:**

The purpose was to investigate prevalence of bilateral discoid lateral menisci (DLM) in Han Chinese patients who received surgery for symptomatic DLM, as well as a follow-up study of their asymptomatic contralateral knees using magnetic resonance imaging (MRI).

**Methods:**

A total of 110 patients [50 males and 60 females; average age: 21.95 ± 12.77 years (range: 6 to 67 years)] admitted to our hospital with symptomatic DLM were treated with arthroscopic surgery. The contralateral asymptomatic knees were evaluated for DLM by MRI. Postoperative clinical evaluation was performed using the Lysholm knee scoring scale and International Knee Documentation Committee subjective knee evaluation.

**Results:**

Eighty (72.73 %) of 110 symptomatic DLM patients had bilateral DLM, of which 68 (85 %) were of homotype (same type). Fourteen of 80 bilateral DLM patients were symptomatic and received operations in both knees. Twelve of remaining 66 bilateral DLM patients with asymptomatic one knee underwent a second arthroscopic surgery as their asymptomatic knees became symptomatic over the five-year interim. Of these 12 cases, seven exhibited no shift and five showed posterocentral meniscal shift. Furthermore, at least two cases showed progression from asymptomatic grade II to symptomatic grade III over the interim. All patients showed significant improvement after surgery.

**Conclusions:**

The bilateral DLM rate of Han Chinese patients with symptomatic DLM was relatively high at 72.7 %, and 85 % of those were of homotype.

## Background

The most common anatomic variant of the knee joint meniscus is the discoid meniscus [1]. First discovered by Young in 1889, discoid menisci, when present, are almost always located on the lateral aspect of the knee [2], and medial discoid menisci are relatively rare. Discoid lateral meniscus (DLM) is a common congenital disorder which not only affects the morphology and movement of the meniscus, but can also change the mechanical relationship of the knee, resulting in injury [1, 3, 4]. According to clinical data, the prevalence of DLM varies greatly depending on the population studied. The reported prevalence of DLM ranged from 0.4 % to 5 % in Europe, but was much higher in parts of Asia (approximately 5.8 % to 17 %) [5–9].

Although a large body of literature exists regarding the prevalence of DLM in different populations, few have reported the prevalence of bilateral DLM. This is primarily due to the high proportion of asymptomatic DLM patients, making it difficult to assess the actual frequency of bilateral DLM. Several clinical studies have reported bilateral DLM prevalence of between 5 and 20 % in their respective populations [6, 10, 11]. Among studies conducted in East Asia, Kato et al. used cadaveric knees to study the prevalence of lateral discoid menisci in the Japanese population [12]. They found that bilateral menisci were of homotype (the same shape) in knees of 253 of 279 (91 %) Japanese cadavers [12], and South Korean studies reported the prevalence of bilateral DLM to be between 79 % and 97 % for patients with unilateral symptomatic DLM [3, 13].

Arthroscopy has long been the gold standard in the diagnosis of knee abnormalities, but MRI is rapidly becoming a favored alternative because it is non-invasive and cost-effective [14–16]. The widespread application of MRI has made the diagnosis of discoid meniscus simpler. In addition to its routine use in examining affected knees’ menisci, MRI can be used in the follow-up examination of the contralateral knees when they are suspected of developing bilateral DLM.

The purpose of this study was to investigate the prevalence of bilateral DLM in Han Chinese patients who underwent arthroscopic surgery for symptomatic DLM, and to classify the types of DLM morphology by MRI. In addition, we hypothesized that there might be a role for prophylactic management of asymptomatic DLM knees, and have performed a surveillance of up to five years to assess and treat contralateral asymptomatic knees of those bilateral DLM patients that have developed symptoms, in an attempt to determine the incidence and patterns of disease progression based on MRI findings.

## Methods

### Subjects

A total of 121 symptomatic DLM (in one or both knees) patients underwent arthroscopic surgery at our hospital (Six People’s Hospital of Shanghai Jiao Tong University) from January 2009 to April 2010. All affected knees were examined with MRI prior to hospital admission. Patients with symptoms in both knees and who had at least one knee diagnosed and treated previously for DLM, or patients with symptomatic DLM in one knee (and amenable to MRI examination of the contralateral knee) were included in the study. Patients who were not ethnic Han Chinese were excluded from the study. Based on these criteria, 110 patients were enrolled in this study. All knees were evaluated by physical examination and clinical scoring including the Lysholm knee scoring scale and International Knee Documentation Committee (IKDC) subjective knee evaluation. Functional assessments (including Lysholm knee scores and IKDC scales) were performed both before and after surgery. All MRIs were performed prior to surgery.

All symptomatic DLM knees were treated with arthroscopic surgery. The contralateral knees of patients with unilateral DLM were examined by MRI to ascertain if DLM was present in the contralateral knee. A morphological analysis of all DLMs was also performed . All symptomatic DLM knees were diagnosed by MRI and confirmed by arthroscopy. All contralateral DLMs were also diagnosed by MRI scanning.

### MRI scanning and typing of DLMs

All images were acquired on a 1.5 T Signa system (GE Healthcare, USA). Diagnostic criteria for DLM included: (1) anterior and posterior horns of the meniscus connected to form a tie-like configuration in more than three contiguous sagittal 5-mm-thick slices; (2) the presence of a meniscal body greater than 15 mm wide or extending into the intercondylar notch on coronal images, and the ratio of meniscal body to the area of lateral tibial plateau greater than 50 % [6, 17]. If a meniscus covered the entire lateral tibial plateau on coronal images, it was defined as a complete DLM; if the lateral tibial plateau was not completely covered, the meniscus was considered an incomplete DLM [13, 14].

All MRI images were evaluated by two experienced musculoskeletal radiologists and a sports medicine expert. If MRI readings differed among the three physicians, a consensus was reached among the three physicians after detailed discussion.

Based on Crues’ grading system for MRI signals, the meniscal signals were classified into one of the following grades: 0, normal meniscus, uniform low signal and regular in shape; I, increased focal signal shadow of oval or spherical shape but not adjacent to the articular surface of the meniscus; II, increased horizontal and linear meniscal signals that did not reach the margins of the meniscus; III, high intra-meniscal signal that reached the articular surface of the meniscus [18].

**Fig. 1:**
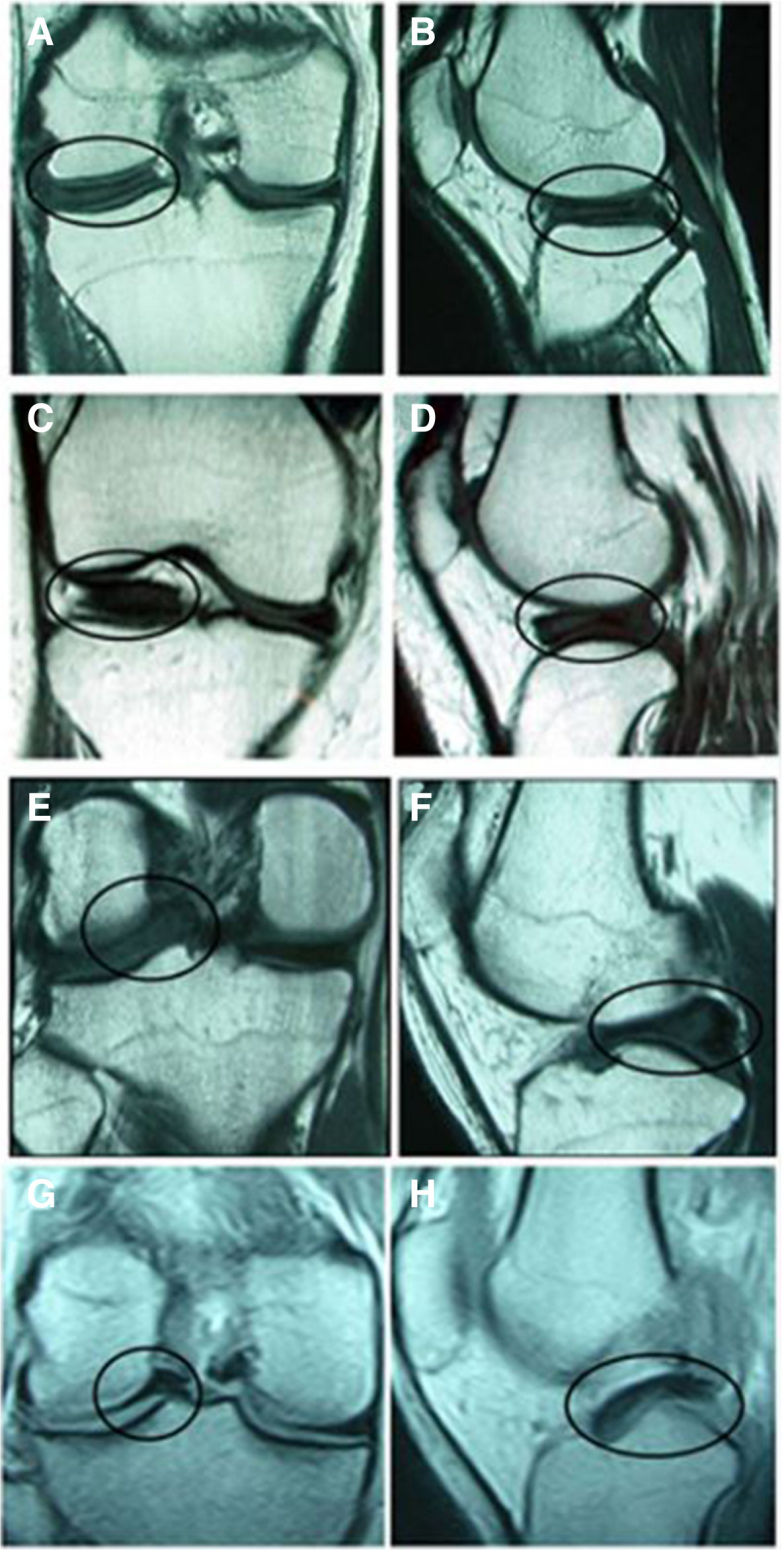
T1-weighted MR images of DLM with different types of shifts. Coronal (**a**) and sagittal (**b**) images of no shift, coronal (**c**) and sagittal (**d**) images of anterocentral shift, coronal (**e**) and sagittal (**f**) images of posterocentral shift and coronal (**g**) and sagittal (**h**) images of central shift

According to the MRI classification method proposed by Ahn et al., each lateral meniscus was classified as one of three types: normal type (NM), incomplete discoid type (ICDM), or complete discoid type (CDM)[13]. In addition, according to the MRI injury classification proposed by Ahn et al. [19]., each DLM was further classified as one of four types of meniscal injury, i.e., no shift, anterocentral shift, posterocentral shift, or central shift (Fig. 1).

This study was approved by the Office of Research Ethics of Shanghai Sixth People’s Hospital of Shanghai Jiao Tong University. All adult patients or parents/legal guardians of patients under the age of 18 gave their written informed consent prior to participation in the study. All patients treated at our hospital gave their informed consents that their medical records might be used for research purposes and for future publications.

### Postoperative follow-up

Patients were followed up at 1, 3, 6, 12, 18 and 24 months after surgery in the clinic or by telephone. The Lysholm scale and IKDC evaluation form of each patient were recorded and analyzed.

### Statistical analysis

All statistical analyses were performed using SPSS 15.0 for Windows (SPSS Inc., Chicago, IL, USA). The data before and after surgery were compared using the paired *t*-test. A p-value <0.05 was considered statistically significant.

## Results

### Patient characteristics

A total of 110 symptomatic DLM patients [50 males (45.45 %) and 60 females (54.55 %), with an average age of 21.95 ± 12.77 years (range: 6 to 67 years)] who received arthroscopic operation(s) were included in this study (Table 1). Patients < 18 years of age accounted for 47.3 % of cases. Among these 110 symptomatic DLM patients who received surgery, 54 cases involved only the left knees, 30 cases involved only the right knees, 14 were bilaterally symptomatic DLM patients who received operations in both knees, and there were additional 12 patients with surgery in one knee who later developed symptoms in the contralateral knees (Table 1).

**Table 1:** Demographics information for 110 symptomatic DLM patients.

Variables	Statistics
Age, yrs [mean ± SD (range)]	21.95 ± 12.77(6,67)
<18 [count (%)]	52 (47.3)
≥18 [count (%)]	58 (52.7)
Gender [count (%)]	
Male	50 (45.5)
Female	60 (54.5)
DLM locations [count (%)]	
Left	54 (49.1)
Right	30 (27.3)
Bilateral	14 (12.7)
Bilateral*(successive)	12 (10.9)
Lysholm knee scoring scale [mean ± SD (range)]	77.75 ± 10.77(45,89)
IKDC score [mean ± SD (range)]	74.35 ± 10.41(40,88)
Follow-up duration, months [mean ± SD (range)]	22.04 ± 2.83 (18,24)

DLM, discoid lateral meniscus; IKDC, International Knee Documentation Committee.

*These patients had prior surgery for DLM on the other knee at time of assessment.

All patients were followed-up in the clinic (91 %) or by telephone (9 %). The duration of follow-up ranged from 18 to 24 months (average follow-up time: 22.04 months), starting from the first operation. No complication, including wound or joint infections, was observed. Overall Lysholm knee scores and IKDC scale were 77.75 ± 10.77 and 74.35 ± 10.41, respectively.

### Homotype rate and frequency of bilateral DLM

According to Ahn’s MRI classification [19], and the results of the MRI scanning, 80 (72.73 %) of the 110 patients had bilateral DLM (Table 2), of which 68 (85 %) were homotypes (same type of either CDM/CDM or ICDM/ICDM) and the rest were heterotypes (CDM/ICDM). In addition, among 110 symptomatic DLM patients there were 80 bilateral DLM patients (72.73 %) based on MRI scanning, of which 14 patients were symptomatic and received surgery for both knees; of the remaining 66 patients who had only symptoms in one knee at time of their first arthroscopic surgery, 12 eventually developed symptoms and received a second surgery in the contralateral knees over the five-year interim. The time span between the first and second surgeries in those 12 patients was as follows: two cases within 1 year, seven cases within 1–2 years, and three cases within 3–5 years (Tables 3 and 4).

**Table 2:** Morphology, MRI typing and surgical treatment of DLM

	Count (%)
Morphology of Bilateral DLM (80 patients)	
Homotype DLM	
CDM/CDM	61(76)
ICDM/ICDM	7(9)
Heterotype DLM (CDM/ICDM)	12(15)
Surgery & MRI type (total 136 knees)	
Central partial meniscectomy	
No shift	44(32.35)
Anterocentral shift	1(0.7)
Posterocentral shift	8(5.9)
Central shift	3(2.2)
Central partial meniscectomy + repair	
Non shift	13(9.6)
Anterocentral shift	1(0.7)
Posterocentral shift	9(6.6)
Central shift	5(3.7)
Subtotal meniscectomy	
No shift	24(17.6)
Anterocentral shift	1(0.7)
Posterocentral shift	10(7.4)
Central shift	17(12.5)

DLM, discoid lateral meniscus; CDM, complete discoid type; ICDM, incomplete discoid type; MRI, magnetic resonance imaging.

**Table 3:** Details of first surgery in symptomatic knees of 12 bilateral DLM patients

			Previous symptomatic knees					
ID	Sex	Age	Site	Surgery date	Surgical treatment	Operation type	MRI grade	Shift type
1	M	19	L	07/2007	Subtotal meniscectomy	Complete	III	Posterocentral
2	F	12	R	02/2009	Meniscoplasty + repair	Complete	III	Central
3	M	13	R	07/2008	Meniscoplasty	Complete	III	No shift
4	F	36	R	01/2008	Meniscioplasty	Complete	I	No shift
5	M	17	L	08/2006	Meniscoplasty	Incomplete	III	posterocentral
6	F	12	R	03/2010	Meniscoplasty	Complete	III	No shift
7	M	17	R	07/2007	Subtotal meniscectomy	Complete	III	No shift
8	M	17	L	01/2008	Subtotal meniscectomy	Complete	II	No shift
9	M	41	L	12/2008	Meniscoplasty	Complete	II	No shift
10	M	17	R	01/2005	Subtotal meniscectomy	Complete	III	No shift
11	M	67	L	02/2009	Subtotal meniscectomy	Complete	II	No shift
12	F	7	L	12/2009	Meniscoplasty	Complete	III	Posterocentral

L, left knee; R, right knee; DLM, discoid lateral meniscus.

**Table 4:** Details of second surgery in contralateral knees of 12 bilateral DLM patients that became symptomatic within 5 years after first surgery.

			Contralateral asymptomatic knees that developed symptoms within a 5-yr post-op period							
ID	Sex	Age	Site	Surgery date	MRI grade (no apparent symptoms)	Surgical treatment	Operation type	MRI grade (at onset of symptoms)	Shift type	Time interval between two operations (months)
1	M	19	R	01/2009	NR	Subtotal meniscectomy	Complete	III	Posterocentral	18
2	F	12	L	09/2009	III	Subtotal meniscectomy	Complete	III	Posterocentral	7
3	M	13	L	07/2009	NR	Meniscoplasty	Complete	III	Posterocentral	12
4	F	36	L	01/2010	NR	Meniscoplasty	Complete	I	No shift	24
5	M	17	R	02/2010	III	Meniscoplasty	Incomplete	III	Posterocentral	42
6	F	12	L	09/2011	III	Meniscoplasty	Complete	III	No shift	18
7	M	17	L	06/2010	NR	Meniscoplasty	Complete	III	Posterocentral	35
8	M	17	R	09/2009	NR	Meniscoplasty	Complete	III	No shift	20
9	M	41	R	11/2009	NR	Subtotal meniscectomy	Complete	III	No shift	11
10	M	17	L	01/2010	NR	Meniscoplasty + repair	Complete	III	No shift	60
11	M	67	R	01/2010	II	Subtotal meniscectomy	Complete	III	No shift	11
12	F	7	R	07/2011	II	Meniscoplasty	Incomplete	III	No shift	19

L, left knee; R, right knee; DLM, discoid lateral meniscus; NR, not recorded.

### Surgical treatment and MRI evaluation of the symptomatic knees in 12 bilateral DLM patients

MRI classification and surgical treatment of the symptomatic knees in 12 bilateral DLMs are summarized in Table 3. Among them, eight knees had no shift and three knees had posterocentral shift. In an attempt to retain as much of the menisci during surgery as possible to prevent progression to osteoarthritis [20], we carried out subtotal meniscectomy in five of these 12 cases (Table 3).

According to the MRI grading system proposed by Crues et al. [18], eight of 12 symptomatic knees presented as grade III signal, three as grade II signal, and one as grade I signal (Table 3). The one patient who had grade I signal was operated on because, although the patient’s knee was grade I, the discoid meniscus was a complete type and the knee was symptomatic and affecting the patient’s quality of life, therefore, surgery was performed in this patient.

### Surgical treatment and MRI evaluation of the contralateral knees in 12 bilateral DLM patients

MRI classification and surgical treatment of the contralateral knees in 12 bilateral DLMs were summarized in Table 4. Among them, seven knees had no shift and five knees had posterocentral shift. Subtotal meniscectomy was carried out in four of 12 cases.

According to the MRI grading system proposed by Crues et al. [18], of the 12 bilateral DLM cases which underwent a second arthroscopic surgery on the contralateral (initially asymptomatic) knee within five years of the first surgery, 11 of 12 knees presented as grade III signal (Table 4). In addition, at least two of the 12 knees showed progression of disease from grade II to grade III (Table 4).

### Postoperative evaluation grouped according to age

The recovery of both adolescent and adult patients was satisfactory after 24 months of follow-up. For the adolescents, the average Lysholm scores significantly improved from preoperative levels of 78.44 ± 9.12 to 97.00 ± 2.13 at the last follow-up and IKDC scores improved from 74.85 ± 8.79 to 96.08 ± 2.66 (both *P* <0.001; Table 5). Compared to preoperative scores, both postoperative Lysholm scores and IKDC scores for adults also improved significantly (*P* <0.001; Table 5).

**Table 5:** Pre- and post-operative Lysholm and IKDC scores of patients grouped based on age

	Pre-operative	Post-operative	*p*
<18 yrs (n = 52)			
Lysholm knee scoring scale	78.44 ± 9.12	97.00 ± 2.13	<0.001*
IKDC score	74.85 ± 8.79	96.08 ± 2.66	<0.001*
≥18 yrs (n = 58)			
Lysholm knee scoring scale	77.14 ± 12.1	95.69 ± 3.69	<0.001*
IKDC score	73.91 ± 11.74	94.76 ± 3.76	<0.001*

IKDC, International Knee Documentation Committee.

Data were presented as mean ± standard deviation and tested using the paired t-test.

*Indicates a significant difference between the two groups, p <0.05.

## Discussion

Discoid lateral meniscus (DLM) is a common anatomical variant of the knee. The frequency of DLM is low in Europe but relatively high in Asia. Smillie et al. completed 10,000 cases of meniscectomy, 467 cases of which were DLM (4.6 %) [21]. Dickason et al. evaluated 14,731 menisci and found 102 discoid lateral menisci in 6,691 lateral menisci (1.5 %) [22].

Kim et al. reported that between 1990 and 1992, 77 of 534 arthroscopy cases (14 %) were diagnosed with DLM [23]. Over a 20-year period in Japan, Ikeuchi noted that the frequency of DLM was 16.6 % on arthroscopic examination [6]. In India, 95 in 1643 knees (5.8 %) were diagnosed with DLM [9]. However, these reports are results of arthroscopy in symptomatic patients, whereas studies of the asymptomatic population are almost absent. To determine the prevalence of asymptomatic DLM in general population, Fukuta et al. examined l15 Japanese asymptomatic volunteers (aged from 13 to 76) using MRI, and reported the frequency of DLM to be 13 %; their study also showed that, at least in Japan, asymptomatic DLM could occur in any age group [24].

To our best knowledge, only a few studies have reported the frequency of bilateral DLM. Bae et al. checked arthroscopic features of the lateral meniscus in asymptomatic contralateral knees in 52 DLM patients who presented with symptomatic DLMs [3]. They reported a frequency of bilateral DLM of 79 % (41 of 52 contralateral knees) and 65 % of patients (34 pairs of knees) had the same DLM types [3]. Ahn et al. examined the contralateral knees of 33 Korean male soldiers with single-knee DLM surgery during the period from 2006 to 2008 [13]. They found that bilateral DLM occurred in 97 % of the patients [13]. Our study may be the first to report its prevalence in the Han Chinese patients.

In clinical practice, we occasionally observed asymptomatic DLM in contralateral knees of symptomatic DLM patients, and their bilateral DLM often were of the same type; studies have also supported this finding. Kato et al. evaluated 279 Japanese cadavers to study the shape of the menisci, and found that 91 % of DLMs were of the same morphology [12]. Therefore, when a patient presents with DLM in one knee, it is important to carefully examine the other knee. From the surgeon’s point of view, preoperative imaging with MRI to assess patients with symptomatic DLM, as well as detecting DLM and other subclinical lesions in the contralateral knee, can be important for preoperative planning. In some cases, it is necessary to confirm the presence of DLM by diagnostic arthroscopy to prevent potential injury. But given that MRI is recognized as a reliable alternative to arthroscopic diagnosis [14–16], patients are now less willing to undergo invasive arthroscopic examination for asymptomatic knees; for this reason, we adopted the noninvasive MRI to examine contralateral knees in Han Chinese patients who underwent arthroscopic surgery. We found bilateral DLMs in 72.7 % (80 of 110 DLM patients) of contralateral knees, only slightly fewer than the 79 % reported by Bae et al. [3]. Minor difference is expected as there appears to be difference in the prevalence of DLM among different populations [5–9].

For symptomatic DLM, most authors recommended arthroscopic meniscoplasty to preserve the meniscus [10, 25–28], while DLM complicated by meniscal tear often leads to partial meniscectomy or repair. It has been suggested that abnormal pressure on bone and cartilage caused by DLM could induce osteochondritis dissecans of the lateral femoral condyle [29]. For asymptomatic discoid meniscus, follow-up surveillance would suffice and there is no need for further treatment unless symptoms appear, as the knee joint may have adapted to the anatomic configuration and can maintain normal function. But the knee with DLM is still at risk of tearing and the DLM may cause abnormal conduction of loads at the knee, so that even without history of trauma, symptoms may still eventually develop in knees with asymptomatic DLM. But it remains unclear whether prophylactic meniscoplasty can reduce these risks with few or no complication [6, 30], as subtotal meniscectomy of asymptomatic DLM after injury has been shown to increase the risk of arthritis or degenerative changes [31, 32]. It’s for this reason that our 24-month follow-up was an attempt to approximate when an asymptomatic discoid meniscus began to exhibit symptoms, so meniscoplasty could be carried out to avoid subtotal meniscectomy.

Based on our MRI results, 80 (72.73 %) of the 110 symptomatic DLM patients had bilateral DLM and 68 were of homotypes. We found that 12 (18 %) of 66 bilateral DLM patients who were initially asymptomatic in one knee underwent a second arthroscopic surgery within five years after the first surgery as the contralateral knees developed symptoms. 11 of these 12 knees presented as grade III signal at time of operation, and at least two of the 12 knees showed progression from grade II to grade III over the interim. Many DLMs are usually asymptomatic clinically, but due to the lack of the wedge-shaped supportive role of the normal meniscus, flexion and rotation of the knee can cause shear forces on the DLM, resulting in degeneration or potential injury. Therefore, MRI signals of grade II and III can be found not only in symptomatic DLMs, but also in asymptomatic DLMs, as shown by our results. The data proved our initial hypothesis that contralateral asymptomatic DLM of patients who underwent unilateral DLM surgery may be at risk for future injury. Further research with longer follow-up time is needed to ascertain if meniscoplasty on the asymptomatic knees, especially those with MRI grade II or above, should be performed at time of arthroscopic surgery on their first symptomatic knees.

The increase of MRI signals which do not reach the upper and lower margins of the meniscus (grade I to II signal change), indicates either pure degeneration or an intrameniscal tear, whereas high signal extending to the upper and lower margins (grade III signal change) represents a dominant tear. These findings were based on the analysis of non-discoid meniscus, and may or may not be applicable for the discoid meniscus [15, 16]. We feel that MRI signal changes of DLM undergoing degeneration or tear would correlate with disease severity more than those of the non-discoid meniscus. In this study, of the 12 bilateral DLM patients that had undergone previous arthroscopic surgery, the asymptomatic knees of five cases initially examined by MRI showed three cases of grade III signal and two cases of going from grade II to grade III changes (Table 4). Although there were no definite tears, we feel that grade III meniscal signal changes may indicate potential injury.

The need for a second surgery on the contralateral DLM in the 12 bilateral DLM patients suggests that such cases require further long-term follow-up for the following reasons: (1) although no clinical symptoms were observed in the contralateral DLM, grade III signal change indicated the existence of potential injury; (2) because muscle strength and flexibility of the joint decline in the rehabilitation phase after knee surgery, inappropriate exercise or other physical activity of the knee may cause compensatory increase in stress on the contralateral asymptomatic DLM knee, thus the contralateral DLM knee is more prone to injury; (3) if the patients engage in specific sports activities such as dancing, football or basketball which involve emergent stops and frequent rotations, they are more likely to be injured. We will extend follow-up of our patients in order to gather more information concerning the reasons for (and frequency of) change from asymptomatic to symptomatic DLM knees and the optimal timing of surgical intervention. Most agree that the meniscoplasty is the best surgical approach for DLM injury [10, 26–28, 32]. Therefore, if a certain MRI type or signal change can be correlated with the second surgery, for example, complete DLM or grade III signal change indicating potential injury, it may be advisable to perform meniscoplasty as an early surgical intervention (at the same time of surgery for the symptomatic DLM) in order to avoid subtotal meniscectomy and preserve the function of the meniscus. Also, if particular types of sport are identified to aggravate DLM injury, more serious damage may be prevented through adjustment of activities when the patient is present with asymptomatic DLM knee with subclinical changes.

Our study had several limitations. It was limited only to symptomatic DLM patients who underwent arthroscopic surgery, and not asymptomatic volunteers in the general population, so the result does not reflect the prevalence of bilateral DLM in the general population. Furthermore, the age distribution of our subjects did not reflect that of the general Han population of China. We also did not study the incidence of newly developed bilateral DLM in patients with prior diagnosis of unilateral DLM. Nevertheless, this study still provided information regarding the MRI assessment of knees with subclinical DLM that may be important in planning the course of their treatment. In addition, we did not address potential bias in MRI imaging evaluation in our study. We were also unable to ensure that all patients returned for a follow-up examination at predesignated or scheduled times, so for such patients, we could only contact then by telephone, and for the part of questionnaire that required physical examination, we could only gather answers through verbal questioning. Therefore, inaccurate answers were possible.

## Conclusion

In conclusion, the bilateral DLM rate of Han Chinese patients with symptomatic DLM was relatively high at 72.7 %, and 85 % of those were of homotype. Our study showed that prophylactic meniscoplasty may be beneficial in a small portion of bilateral DLM patients whose asymptomatic contralateral knees displayed a high-grade MRI abnormality, but more controlled studies are needed to define which group of bilateral DLM patients would most likely benefit from prophylactic meniscoplasty.
